# Expression evaluation of exogenous and endogenous alcohol dehydrogenase genes in transgenic *Arabidopsi*s

**DOI:** 10.3389/fpls.2024.1476754

**Published:** 2025-01-22

**Authors:** Haoqiang Yu, Hong Lv, Fengzhong Lu, Qingqing Yang, Huaming Duan, Wanchen Li, Fengling Fu

**Affiliations:** Maize Research Institute, Sichuan Agricultural University, Chengdu, China

**Keywords:** endogenous gene, enzyme activity, exogenous gene, protein accumulation, transgenics

## Abstract

A lot of endogenous genes, as well as genes from related species, are transformed back into crops for overexpression to improve their corresponding traits. However, almost all of these transgenic events remain at the testing stage. Most of the singular transgenic events of crops approved for commercial release are developed by the transformation and heterologous expression of exogenous genes from distant species. To detect the differences in expression, protein accumulation, and enzyme activity between transformed exogenous and endogenous genes, the coding sequences (CDSs) of the alcohol dehydrogenase (ADH) genes were cloned from dicotyledonous *Arabidopsis*, monocotyledonous maize, and prokaryotic *Escherichia coli*, constructed into expression vector pBI121-cMycNY, and used to transform wild-type *Arabidopsis*, respectively. Three homozygous T_3_ lines with a single integration site were screened for each of the three transformed genes by antibiotic screening, polymerase chain reaction (PCR) identification, and genomic DNA resequencing. Real-time quantitative PCR (RT-qPCR) analysis showed that the relative expression levels of the transformed exogenous *ZmADH* and *EcADH* genes were ten or tens of times higher than that of the transformed endogenous *AtADH* gene. After confirming the encoded proteins of these transformed genes by Western blotting, enzyme-linked immunosorbent assay (ELISA) showed that the accumulation levels of the proteins encoded by the transformed genes *ZmADH* and *EcADH* were significantly higher than that encoded by the transformed endogenous gene *AtADH*. Enzyme reaction assay showed that the ADH activities of the T_3_ lines transformed by the exogenous genes *ZmADH* and *EcADH* were also significantly higher than that transformed by the endogenous gene *AtADH* as well as the wild-type control. These results indicated that exogenous genes were more conducive to transgenic improvement of crops.

## 1 Introduction

Transgenic technology accelerates crop improvement, not only enabling it to acquire biotic and abiotic resistances but also effectively meeting various requirements such as higher yield and nutritional value (Raymond Park et al., 2011; Kamthan et al., 2016). As of 2023, a total of 440 singular transgenic events have been approved for commercial release, and the planting area of genetically modified crops has risen sharply to 20,626 ha, accounting for 80.4%, 73.7%, 32%, 23.8%, and 11.4% of global planting areas of cotton, soybean, maize, rapeseed, and sugar beet, respectively (International Service for the Acquisition of Agri-biotech Applications, 2023). The huge benefits not only come from increased production but also from reduced inputs of pesticides, labor, and machinery (Karalis et al., 2020; Kobayashi et al., 2023).

Of the 472 singular transgenic events approved for commercial release, 332 events were developed by the heterologous expression of transformed exogenous genes, 29 events were transformed by synthetic sequences transcribing antisense or double-stranded RNAs for the interference of disease viruses, and only three events were transformed by the mutated copies of endogenous genes for suppressed expression of undesirable genes (Yu et al., 2022; International Service for the Acquisition of Agri-biotech Applications, 2024). By a strategy of overexpression of endogenous genes, numerous transgenic events have also been developed and reported to improve target agronomic traits, such as the transformation of endogenous genes *SOD* (superoxide dismutase), *VP1* (vacuolar proton-pumping pyrophosphatase), *BADH* (betaine aldehyde dehydrogenase), *LEA* (late embryogenesis-abundant proteins), *DREB* (dehydration-responsive element binding), *NF-Y*, and *WRKY*, in an attempt to improve drought tolerance (Kasuga et al., 1999; Nelson et al., 2007; Xiao et al., 2007; Century et al., 2008; Schilling et al., 2017; Gao et al., 2018). However, all of these efforts remained at the testing stage. Of the five singular transgenic events approved for commercial release with the significant improvement of drought tolerance (International Service for the Acquisition of Agri-biotech Applications, 2024), one was transformed by the exogenous cold shock protein gene *CspB* from genetically distant *Bacillus subtilis* and three by the exogenous choline dehydrogenase genes *BetA* from genetically distant *Escherichia coli* and *Rhizobium meliloti*, respectively, although the endogenous genes of cold shock proteins are also found in many eukaryotic species (Castiglioni et al., 2008; Tollefson, 2011). Only one was transformed by the exogenous transcription factor gene *Hahb-*4 from a sexually incompatible sunflower (*Helianthus annuus*) (Ribichich et al., 2020).

The most successful achievements of transgenic improvement are none other than herbicide and insect resistance of cotton, maize, soybean, canola (*Brassica napus*), and some other crops (Padgette et al., 1995; Bates et al., 2005; Raymond Park et al., 2011; Klumper and Qaim, 2014; Kamthan et al., 2016). The 291 and 241 singular transgenic events approved for commercial release were transformed by exogenous genes from genetically distant species (Thompson et al., 1987; Stalker et al., 1988; Ghareyazie et al., 1997; Castle et al., 2004; Bates et al., 2005; Showalter et al., 2009; Cui et al., 2011; Guo et al., 2015; Karthik et al., 2020; International Service for the Acquisition of Agri-biotech Applications, 2024). The same strategy was successfully applied to the transgenic manipulation of “Golden” rice and potato for biofortification of vitamin A (Ye et al., 2000; Paine et al., 2005; Chitchumroonchokchai et al., 2017; Napier et al., 2019; Yang et al., 2021) and the transgenic improvement of canola for oil quality (Napier et al., 2019; Kinney et al., 2022; International Service for the Acquisition of Agri-biotech Applications, 2024).

In plants, alcohol dehydrogenase (ADH) catalyzes the reversible reduction of aldehydes that is key to the establishment of the fermentative metabolism and to the constant supply of nicotinamide adenine dinucleotide (NADH) (Chung and Ferl, 1999). In response of plants to oxygen shortage under abiotic or biotic stresses such as waterlogging, submergence, saline-alkaline, low temperature, and pathogen infection, the fermentative metabolism allows ATP production when mitochondrial respiration is hampered (Millar and Dennis, 1996; Shi et al., 2017; Song et al., 2019; Su et al., 2020; Ventura et al., 2020; Zhou et al., 2020; Shen et al., 2021). In the *Arabidopsis* and maize genome, the *ADH* gene exists as a single copy and double copies, respectively (Chang and Meyerowitz, 1986; Peters and Frenkel, 2004). On the basis of constitutive expression, their expression is also induced by various stress conditions such as oxidation, dehydration, and low temperature through signal transduction pathways such as abscisic acid (Dolferus et al., 1994; Peters and Frenkel, 2004). In the genome of *Escherichia coli*, the *ADH* gene exists as a single copy (Blattner et al., 1997). The activity of ADH is easy to be detected by using commercial kits.

In this study, the CDSs of the *ADH* genes were cloned from *Arabidopsis*, maize, and Escherichia coli and transformed into wild-type *Arabidopsis*, which has only a single copy of the *ADH* gene in its genome. Homozygous T_3_ lines with a single integration site of the T-DNA were screened and used to detect the differences in relative expression levels, protein accumulation, and enzyme activity, in order to provide reference for strategy choice of transgenic improvement of crops.

## 2 Materials and methods

### 2.1 CDS cloning and transformation

The CDSs of the *ADH* genes were amplified from the cDNA samples of wild-type *Arabidopsis* (Col-0) and maize inbred line B73, as well as a genomic DNA sample of *E*. *coli* with homologous recombination primers (**Supplementary Table S1**), separated by 1% agarose gel electrophoresis, purified using the Universal DNA Purification Kit (Tiangen, Beijing, China), and constructed into dicotyledonous expression vector pBI121-cMyc-NY (**Supplementary Figure S1**) using homologous recombination kit ClonExpress II (Vazyme, Nanjing, China), respectively. The recombined vectors were transformed into competent cells, Trans-T1 of *E*. *coli* (TransGen, Beijing, China), incubated in LB liquid medium, and screened on LB plates containing 50 mg/l kanamycin. The vector plasmids were purified using the TIANprep Mini Plasmid Kit (Tiangen, Beijing, China) and then used to transform *Agrobacterium tumefaciens* competent cells Gv3101-pSoup-19 (Angyubio, Shanghai, China). After screening on LB plates containing 50 mg/l kanamycin and rifampicin and identification by bacterial PCR and sequencing at Kangyou Biotech (Hangzhou, China), the positive monoclonals were incubated in LB liquid medium, recovered, and used to transform wild-type *Arabidopsis* (Col-0) by floral dipping. Homozygous T_3_ lines were screened on MS plates containing 50 mg/l kanamycin, identified by PCR amplification using the same primers as shown in **Supplementary Table S1**, and calculated for transformation rate.

### 2.2 Identification of T-DNA integration sites

The genomic DNA of each homozygous T_3_ line was extracted using CTAB buffer. After detection for concentration and purity in an NanoDrop 1000 spectrophotometer (Thermo Fisher Scientific Ltd., Paisley, United Kingdom) and identification for integrity by 1% agarose gel electrophoresis, the samples were resequenced (10 × deep) on a DNBSEQ-T7 platform at Gene^plus^ (Shenzhen, China). After preliminary evaluation of the raw data for quality, clean reads were filtered by the FASTP software (https://github.com/OpenGene/fastp) and aligned against the sequences of the *Arabidopsis* genome TAIR10 (http://www.arabidopsis.org) and the expression vector pBI121-cMyc-NY to find out the integration sites of T-DNA. As described by Zhang et al. (2011), specific primers (**Supplementary Table S2**) were designed using online software Primer-BLAST (https://blast.ncbi.nlm.nih.gov) and used to amplify the flanking sequences of the left (from 1000 bp upstream of the integration sites to 1000 bp of the T-DNA) and right (from 1000 bp of the T-DNA to 1000 bp downstream of the integration sites) borders of the T-DNA integration sites from the DNA samples extracted as above from each of the homozygous T_3_ lines. The amplified products were separated by 1% agarose gel electrophoresis, recovered, and sequenced at Kangyou Biotech (Hangzhou, China). The sequenced results were aligned against the sequences of the *Arabidopsis* genome TAIR10 (http://www.arabidopsis.org) and the expression vector pBI121-cMyc-NY again for confirmation of the number and integration sites of T-DNA.

### 2.3 Expression detection of transformed genes

Three biological replates of seedlings of the homozygous T_3_ lines and their type wild control were incubated in a phytotron for 30 days. Consistent plants were sampled from each replicate. RNA was extracted from one-third of each sample using RNAiso Plus (TaKaRa, Dalian, China) and reverse transcribed into cDNA using ABScript Neo RT Master Mix for qPCR with gDNA Remover (ABclonal, Wuhan, China). Specific primers (**Supplementary Table S3**) were designed using online software Primer-Blast (https://blast.ncbi.nlm.nih.gov) and used for real-time quantitative PCR (RT-qPCR) amplification of the transformed genes *AtADH*, *ZmADH*, and *EcADH*, as well as the reference gene *AtUBQ5* using the Universal SYBR Green Fast qPCR Mix (Abclonal, Wuhan, China) in a CFX96 MangerTM Real-Time System (Bio-Rad, CA, USA) with four technical replicates. The relative expression levels of the three transformed genes were calculated, normalized, and analyzed for statistical significance using the 2^−ΔΔCT^ method of the CFX96 Manger^™^ software v 2.0.

### 2.4 Western blotting

Total protein was extracted from another one-third of each of the T_3_ seedling samples using the Plant Total Protein Extraction Kit (Coolaber, Beijing, China), detected for concentration using the BCA Protein Assay Kit (Coolaber, Beijing, China), and diluted to gradient standard samples with the extraction buffer. The OD_562_ of the gradient standard samples were detected in a microplate reader Varioskan LUX (Thermo Fisher Scientific Ltd., Paisley, United Kingdom) and used to establish the standard curve. The concentration of total protein in the samples was calculated and diluted to 5 μg/μl with the extraction buffer. Half of each of the diluted protein samples were separated by 10% sodium dodecyl sulfate-polyacrylamide gel electrophoresis (SDS-PAGE) using the SDS-PAGE Gel Preparation Kit (Biosharp, Hefei, China) and transferred onto a polyvinylidene fluoride membrane (Millipore, Billerica, USA). After blocking and rinsing, the membrane was incubated in Myc-Tag pAb antibody (Abclonal, Wuhan, China) solution at 4°C for 3 h, incubated in anti-rabbit IgG (H+L) antibody (Jackson, MS, USA) solution at room temperature for 30 min, rinsed with rinsing buffer for 3 × 10 min, colored using Ultra-sensitive ECL Chemiluminescent Substrate Kit (Powerful Biology, Wuhan, China), and imaged in Gel Doc™ EZ System (Bio-Rad, Hercules, CA, USA).

### 2.5 Enzyme-linked immunosorbent assay

The ADH standard sample of the ADH ELISA Kit (MLBio, Shanghai, China) was diluted to gradient standard samples and added into the wells of a 96-well plate with three technical replicates. The other half of each diluted protein sample was added into the other wells of the same plate with three replicates. One hundred microliter of HRP antibody was added into each of these wells. The plate was sealed with film, incubated at 37°C for 1 h, and dried by removing the solution in the wells. The wells were washed 5 times by being filled with the diluted washing buffer, incubated at room temperature for 1 min, and dried by removing the solution, added with 50 μl of 0.01 H_2_O_2_ and 0.1% tetramethylbenzidine, shaken well, incubated at 37°C in the dark for 15 min, added with 50 μl termination solution, shaken well, and read for OD_450_ in a microplate reader Varioskan LUX (Thermo Fisher Scientific Ltd., Paisley, United Kingdom). A standard curve was established based on the concentration of the standard samples and their OD_450_ and used to calculate the concentrations of the enzyme proteins. Due to the different molecular weights of the AtADH, EcADH, and ZmADH proteins, the concentrations were divided by their molecular weight to convert to the accumulation of each enzyme in ng/ml/kDa.

### 2.6 Enzyme reaction assay

The other one-third of each sample was ground in liquid nitrogen, transferred into an Enppendoff tube, weighed (W), added with 1 ml of the extraction solution of the ADH reaction assay kit CAS:9031-72-5 (Solarbio, Beijing, China), incubated on ice for 20 min, and centrifuged at 4°C and 16000 r/min for 20 min. The supernatant was used to be detected for ADH activity (U/g) using the same assay kit in an ultraviolet spectrophotometer UV8000 (Shimadzu, Japan). In the reversible reaction between ethanol and acetaldehyde, ADH catalyzes the reduction of acetaldehyde by NADH to produce ethanol and nicotinamide adenine dinucleotide (NAD^+^). NADH has an absorption peak at 340 nm, while NAD^+^ does not. According to the manual of the assay kit, the ADH activity was calculated as


$$\begin{array}{l} \begin{array}{c} ADH\;\left(U/g\;\right) \end{array}=\frac{\begin{array}{c} \left(\Delta A_{340}\;of\;sample\;tube–\Delta A_{340}\;of\;empty\;tube\right)\times V_{Reaction\;total}\times T\times 10^{6} \end{array}}{\begin{array}{c} d\times \epsilon \times \left(W\times V_{Sample}÷V_{Sample\;total}\right) \end{array}}\\ \;=\frac{\begin{array}{c} 1.61\times \left(\Delta A_{340}\;of\;sample\;tube–\Delta A_{340}\;of\;empty\;tube\right) \end{array}}{\begin{array}{c} W \end{array}} \end{array}$$

Where, *Δ*A_340_ was the difference of absorbance at 340 nm before and after reaction, ϵ (molar extinction coefficient of NADH) = 6.22 × 10^3^ l/mol/cm, d (optical diameter of colorimetric dish) = 1 cm, V_Reaction total_ (total volume of reaction) = 1 ml, V_Sample total_ (the added volume of the ADH extraction solution) = 1 ml, T (reaction time) = 1 min, V_Sample_ (the added volume of the supernatant) = 100 μl, and W was the weight (g) of the ground sample.

## 3 Results

### 3.1 Homozygous T_3_ lines and T-DNA integration sites

**Figure 1:**
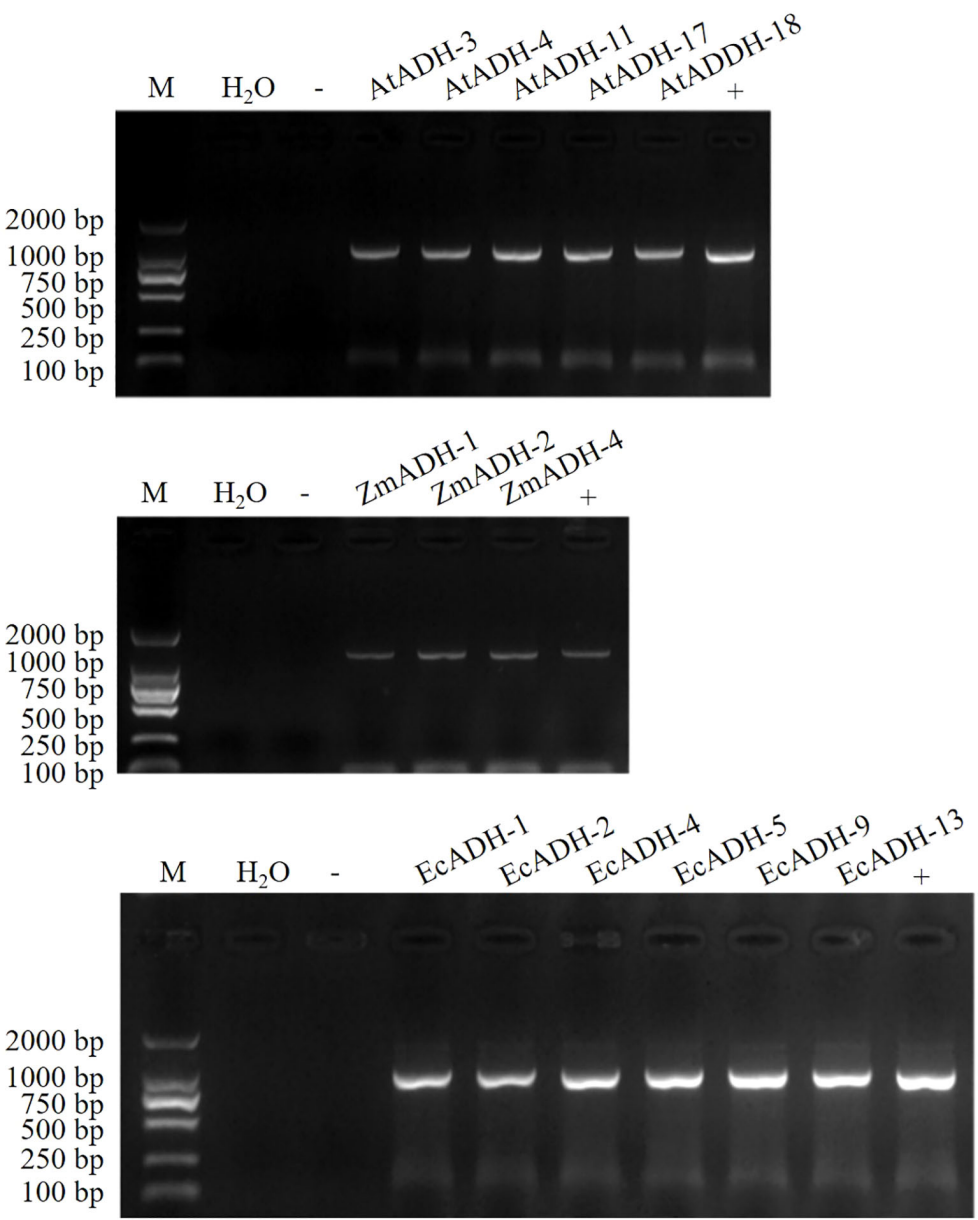
PCR identification of homozygous T_3_ lines. M: DNA marker DL2000, -: Negative control (Wild type), +: Positive control (pBI121-cMyc-NY), the other lanes: amplified CDSs of genes *AtADH*, *ZmADH*, and *EcADH*, respectively, from the T_3_ lines.

The results of the PCR amplification showed that the CDSs of the *AtADH*, *ZmADH*, and *EcADH* genes were successfully cloned (**Supplementary Figure S2**), conducted into dicotyledonous expression vector pBI121-cMyc-NY, and transformed into *Agrobacterium tumefaciens* (**Supplementary Figure S3**). After transformation, 1.2%, 1.4%, and 1.3% T_0_ plants were screened on the resistant plates (**Supplementary Figure S4A**). Each of these T_1_ lines is segregated in a 3:1 ratio (**Supplementary Figure S4B**). Untill T_3_ generation, five, three, and six homozygous lines transformed by the *AtADH*, *ZmADH*, and *EcADH* genes, respectively, were screened on the resistant plates (**Supplementary Figure S4C**) and identified by the PCR amplification (**Figure 1**).

**Figure 2:**
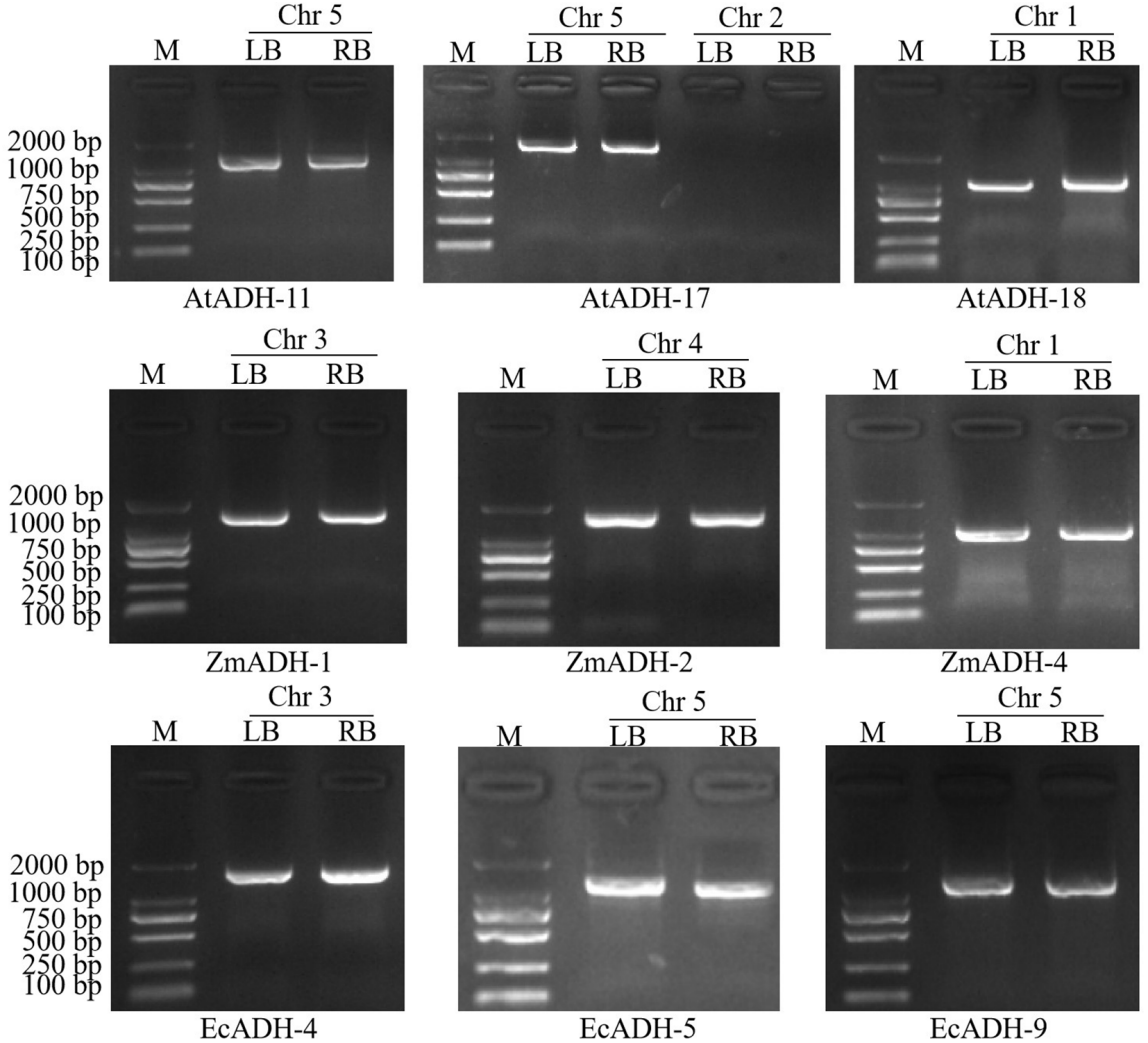
Agarose electrophoresis of amplified products of T-DNA flanking sequences in T_3_ lines. M: DNA marker DL2000, Chr: chromosome, LB: left border, RB right border.

By resequencing the genomic DNA of these 14 T_3_ lines and alignment against the sequences of the *Arabidopsis* genome and T-DNA, a single T-DNA integrated site was found on chromosomes 5, 5, 1, 3, 4, 1, 3, 5, and 5 of lines AtADH-11, AtADH-17, AtADH-18, ZmADH-1, ZmADH-2, ZmADH-4, EcADH-4, EcADH-5, and EcADH-9, respectively. Each of the integrated sequences contained a complete expression structure of 4404, 4024, 2778, 4500, 13131, 4279, 13124, 11887, and 13114 bp, respectively (**Supplementary Table S4**). By using the primers (**Supplementary Table S2**), the sequences flanking the left or right borders, or both, of these integration sites were successfully amplified, sequenced, and aligned against the T-DNA sequences of the expression vector and the *Arabidopsis* genome with high confidence (E value ≤ 2.90E-40 and similarity ≥ 93.60%) (**Supplementary Table S5**). Single integrated sites of the T-DNA containing each of the transformed genes *AtADH*, *ZmADH*, and *EcADH* were confirmed on chromosome 5 of line AtADH-11, chromosome 5 of line AtADH-17, chromosome 1 of line AtADH-18, chromosome 3 of line ZmADH-1, chromosome 4 of line ZmADH-2, chromosome 1 of line ZmADH-4, chromosome 3 of line EcADH-4, chromosome 5 of line EcADH-5, and chromosome 5 of line EcADH-9, respectively (**Figure 2**). These nine homozygous T_3_ lines were used for the following detections.

### 3.2 Relative expression levels of transformed genes

**Figure 3:**
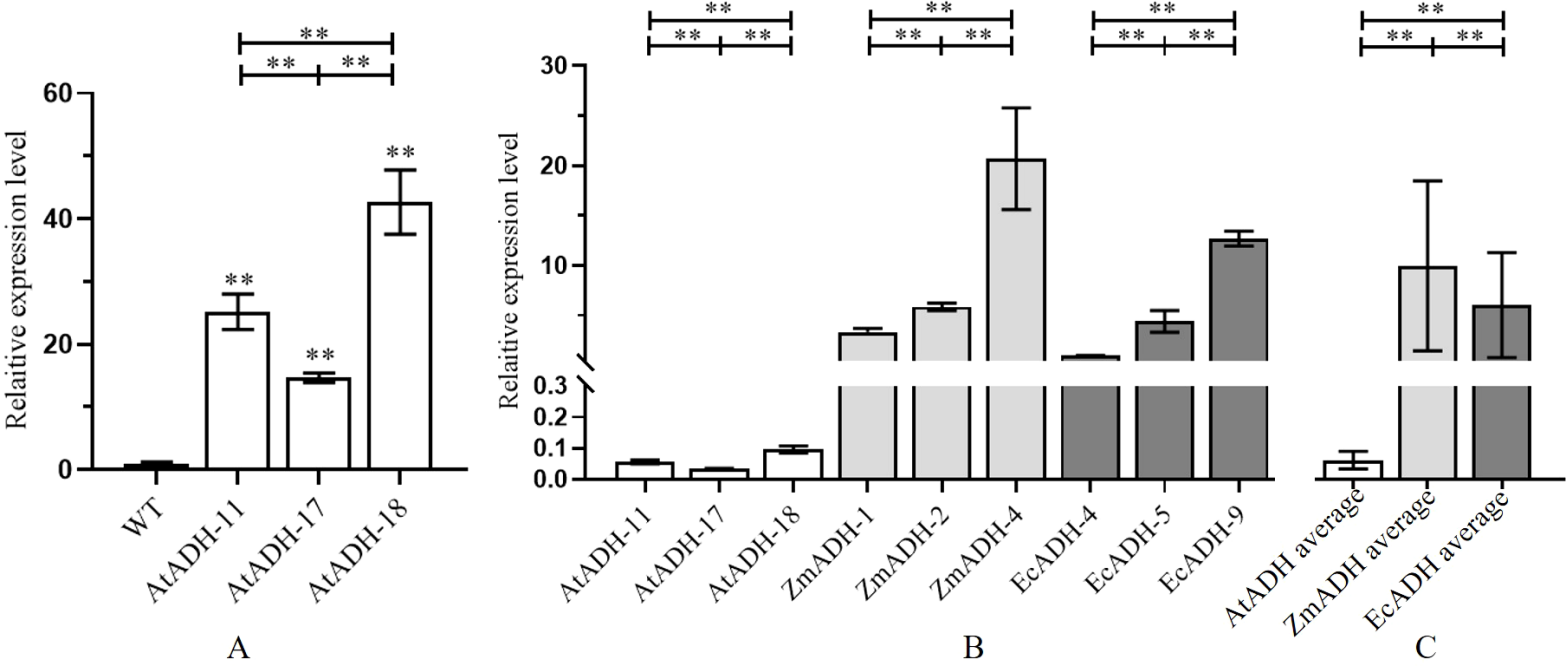
Relative expression levels of transformed genes *AtADH*, *ZmADH*, and *EcADH* in homozygous T_3_ lines. **(A)** The expression level of *AtADH* in overexpressing *AtADH* lines. **(B)** Comparison of relative expression level of *AtADH*, *ZmADH*, and *EcADH* in each line. **(C)** Average relative expression level of *AtADH*, *ZmADH*, and *EcADH* in transgenic lines. The dark, white, dark gray, and light gray columns represent the wild-type control, and the T_3_ lines transformed by *AtADH*, *ZmADH*, and *EcADH*, respectively. The double asterisk (**) at the top of the figure indicates significant differences at *p* < 0.01 among the three T_3_ lines transformed by the same gene and among the average of the three lines transformed by the same gene. The double asterisk (**) at the top of each column indicates significant differences at *p* < 0.01 between each of the T_3_ lines and the wild-type control.

The result of RT-qPCR showed that the transformed endogenous gene *AtADH* was overexpressed under the drive of the *CaMV35S* promoter in all three T_3_ lines (AtADH-11, AtADH-17, and AtADH-18) transformed by the endogenous gene *AtADH*, and the relative expression levels were more than 15 times higher than the wild control with only the endogenous *AtADH* gene (**Figure 3A**). In the three T_3_ lines ZmADH-1, ZmADH-2, and ZmADH-4, and the three T_3_ lines EcADH-4, EcADH-5, and EcADH-9, the transformed exogenous genes *ZmADH* and *EcADH* were overexpressed under the drive of the *CaMV35S* promoter, and their relative expression levels were several or tens of times higher than those in the three T_3_ lines transformed by the endogenous gene *AtADH* (**Figure 3B**). Taken together, the averages of the relative expression levels of the three T_3_ lines transformed by the *ZmADH* gene and the three T_3_ lines transformed by the *EcADH* gene were both tens of times higher than the average of the relative expression levels of the three T_3_ lines transformed by the endogenous *AtADH* gene (**Figure 3C**). All the above differences were greatly significant (**Figure 3**).

### 3.3 Accumulation of proteins encoded by transformed genes

**Figure 4:**
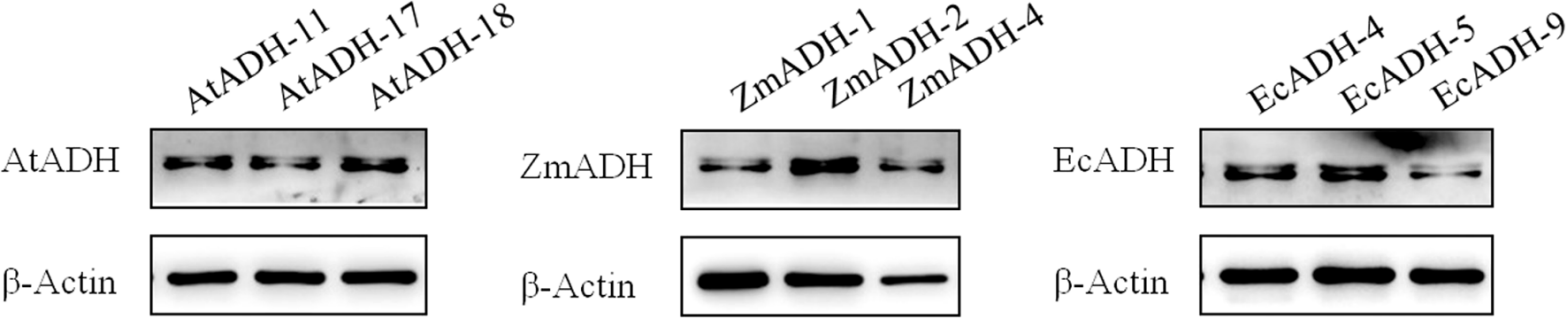
Western blot of AtADH, ZmADH, and EcADH proteins in each of the three T_3_ lines transformed by the *AtADH*, *ZmADH*, and *EcADH* genes, respectively.

**Figure 5:**
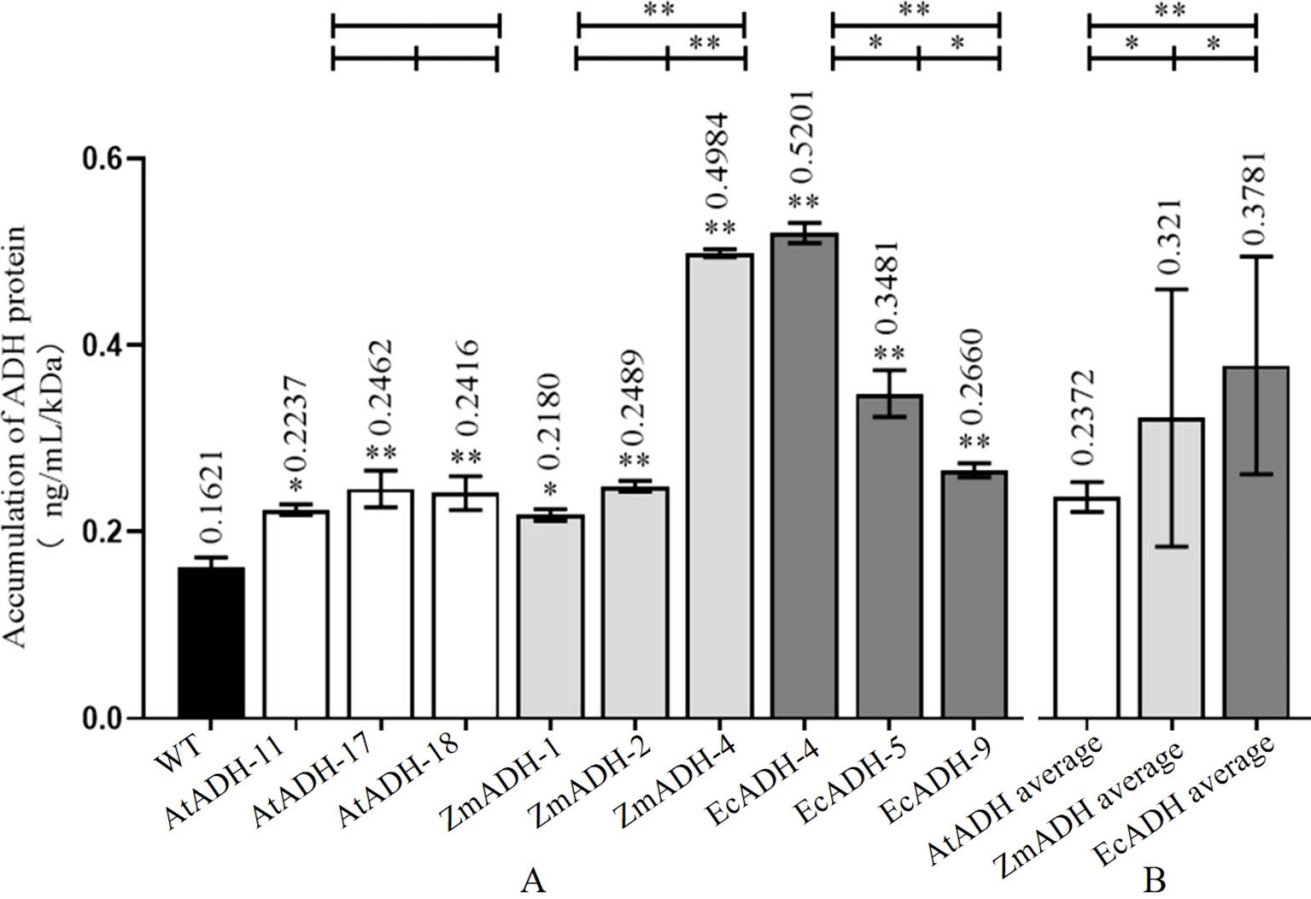
Accumulation of ADH protein in homozygous T_3_ lines. **(A)** ADH amount in each transgenic line. **(B)** Average of ADH amount of different transgenic lines of each gene. The asterisk (*) and double asterisk (**) at the top of each column indicate significant differences between the T_3_ lines and the wild-type (WT) at *p* < 0.05 and *p* < 0.01, respectively. The dark, white, dark gray, and light gray columns represent the wild-type control, and the T_3_ lines transformed by *AtADH*, *ZmADH*, and *EcADH*, respectively. The asterisk (*) and double asterisk (**) at the top of the figure indicate significant differences among the three T_3_ lines transformed by each of the three genes at *p* < 0.05 and *p* < 0.01, respectively.

By Western blotting, the ADH proteins encoded by each of the *AtADH*, *ZmADH*, and *EcADH* genes were detected in the corresponding T_3_ lines with a single integration site of the T-DNA (**Figure 4**). The result of ELISA showed that the accumulation of the ADH protein in the wild-type control was 0.1621 ng/ml/kDa. In the three T_3_ lines (AtADH-11, AtADH-17, and AtADH-18) transformed by the endogenous *AtADH* gene, the accumulation was 0.2237, 0.2462, and 0.2416 ng/ml/kDa, respectively, with no significant difference among them but greatly significantly higher than that in the wild-type control (**Figure 5A**). The accumulation of the ADH protein in the three T_3_ lines (ZmADH-1, ZmADH-2, and ZmADH-4) transformed by the exogenous *ZmADH* gene was 0.2180, 0.2489, and 0.4984 ng/ml/kDa, respectively. The difference between lines ZmADH-1 and ZmADH-2 was not significant, while the difference between these two lines and line ZmADH-4 was significant with a *p*-value less than 0.01. However, all three lines were greatly significantly or significantly higher than that of the wild-type control (**Figure 5A**). The accumulation of the ADH protein in the three T_3_ lines (EcADH-4, EcADH-5, and EcADH-9) transformed by the exogenous *EcADH* genes was 0.5201, 0.3481, and 0.2660 ng/ml/kDa, respectively, with significant or greatly significant differences among them, but all were significantly higher than that of the wild-type control (**Figure 5A**). However, the accumulation of the ADH proteins in each of the six T_3_ lines transformed by the exogenous *ZmADH* and *EcADH* genes, respectively, as well as the average accumulations of the ADH protein in the three T_3_ lines transformed by the exogenous *ZmADH* gene and average accumulation of the ADH protein in the three T_3_ lines transformed by the exogenous *EcADH* genes, were both significantly or greatly higher than the average accumulation of the three T_3_ lines transformed by the endogenous *AtADH* gene (**Figure 5B**).

### 3.4 ADH enzyme activity

**Figure 6:**
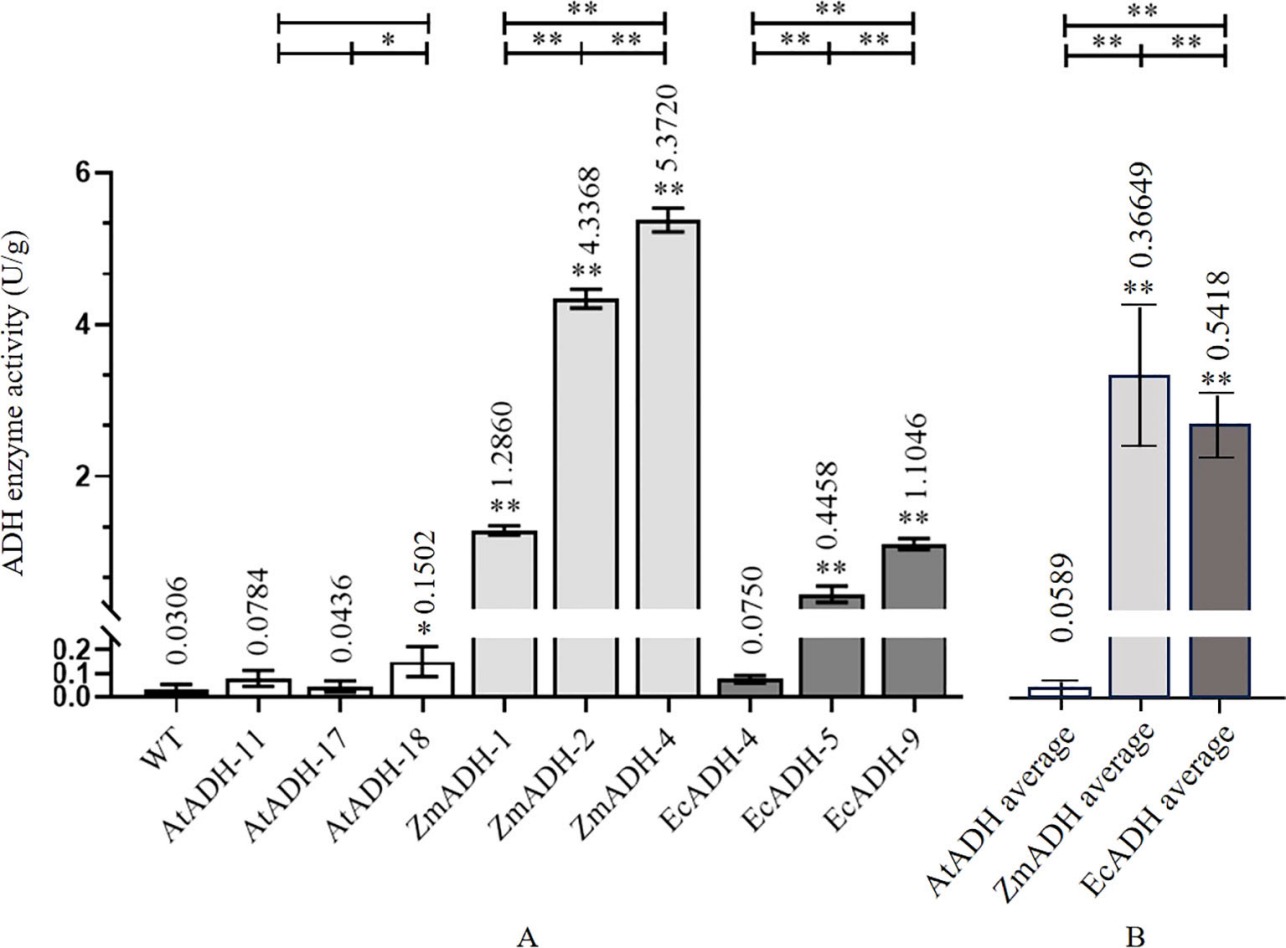
ADH enzyme activity in homozygous T_3_ lines. **(A)** ADH activity in each transgenic line. **(B)** Average of ADH activity of different transgenic lines of each gene. The dark, white, dark gray, and light gray columns represent the wild-type control, and the T_3_ lines transformed by *AtADH*, *ZmADH*, and *EcADH*, respectively. The asterisk (*) and double asterisk (**) at the top of each column indicate significant differences between the T_3_ lines and the wild-type (WT) at *p* < 0.05 and *p* < 0.01, respectively. The asterisk (*) and double asterisk (**) at the top of the figure indicate significant differences among the three T_3_ lines transformed by each of the three genes at *p* < 0.05 and *p* < 0.01, respectively.

In the wild-type control, the activity of the ADH enzyme was as low as 0.0306 U/g. In the three T_3_ lines (AtADH-11, AtADH-17, and AtADH-18) transformed by the endogenous *AtADH* gene, the activities were 0.0784, 0.0436, and 0.1502 U/g, respectively, with only line AtADH-18 significantly higher than that of the wild-type control. However, the activities of the ADH enzyme in the three T_3_ lines (ZmADH-1, ZmADH-2, and ZmADH-4) transformed with the exogenous *ZmADH* gene were 1.2860, 4.3368, and 5.3720 U/g, respectively, with significant differences among them, but all were significantly higher than that of the wild-type control. The activities of the ADH enzyme in the three T_3_ lines (EcADH-4, EcADH-5, and EcADH-9) transformed by the exogenous *EcADH* gene were 0.0750, 0.4458, and 1.1046 U/g, respectively, with greatly significant or significant differences among them. The latter two lines (EcADH-5 and EcADH-9) were significantly higher than the wild-type control. However, the average activity of the ADH enzyme in the three T_3_ lines transformed by the exogenous *ZmADH* gene and the three T_3_ lines transformed by the exogenous *EcADH* gene were significantly higher than the average activity of the ADH enzyme in the three T_3_ lines transformed by the endogenous *AtADH* gene (**Figure 6**).

## 4 Discussion

### 4.1 Less regulation of endogenous mechanisms to the expression of exogenous genes

In the three T_3_ lines transformed by the endogenous *AtADH* gene, the relative expression levels of the transformed genes detected by RT-qPCR inevitably included the expression of the endogenous *AtADH* gene of the recipient wild-type *Arabidopsis*. However, the transformed exogenous *AtADH* gene, driven by the constitutive strong promoter *CaMV35S*, significantly increased their expression levels together with the expression of the endogenous *AtADH* gene. The relative expression levels were more than 15 times higher than those in the wild-type control (**Figure 3A**). In the six T_3_ lines transformed by the exogenous *ZmADH* and *EcADH* genes, the expression levels of the exogenous genes were also significantly increased by ten to tens of times compared to the three T_3_ lines transformed by the endogenous *AtADH* genes (**Figure 3B**). This result indicated that the expression of the transformed exogenous genes was less regulated by endogenous mechanisms and more mRNA could be accumulated, although they were under the drive of the same constitutive strong promoter as the transformed endogenous genes. The same results of database and literature research were also reviewed by Khush (2001); Showalter et al. (2009); Chen et al. (2019), and Yu et al. (2022). The significant differences in the relative expression levels of the transformed genes among the different T_3_ lines transformed with the same gene could be due to the different integration sites of the transformed genes in the *Arabidopsis* genome and their impact on the expression of the transformed genes (**Figure 2**). As we discussed in a previous review (Yu et al., 2022), the transformed exogenous genes overexpress under the control of constitutive promoters and confer recipient crops with novel traits that are usually absent or function in different pathways in the recipient crops themselves. In addition to the influence of the genetic background, growth stage, and environment, the heterologous expression of the transformed exogenous genes in recipient crops is usually less regulated at the transcriptional level (Khush, 2001; Showalter et al., 2009; Chen et al., 2019).

### 4.2 More accumulation of proteins encoded by exogenous genes

By Western blotting, the ADH proteins encoded by each of the transformed *AtADH*, *ZmADH*, and *EcADH* genes were detected in each of the three corresponding T_3_ lines (**Figure 4**). The result of ELISA showed that the accumulation of the ADH protein in the three T_3_ lines transformed by the endogenous *AtADH* gene was significantly higher than that in the wild-type control (**Figure 5A**). The most important was that the accumulation of the ADH proteins in each of the six T_3_ lines transformed by the exogenous *ZmADH* and *EcADH* genes, respectively, as well as the average accumulations of the ADH protein in each of the three T_3_ lines transformed by the exogenous *ZmADH* and *EcADH* genes, respectively, were both significantly higher than the average accumulation of the three T_3_ lines transformed by the endogenous *AtADH* gene (**Figure 5B**). This result indicated that the accumulation of the proteins encoded by exogenous genes was higher than that encoded by endogenous genes. The proteins encoded by the transformed exogenous genes accumulate and function independently of the endogenous metabolic pathways and confer recipient crops with novel or enhanced traits (Adamczyk and Sumerford, 2001; Palma et al., 2014; Melo et al., 2016). In the 13 commercial cotton varieties, the accumulation of the Cry protein was also found to nonlinearly correlate with the heterologous transcription levels of the transformed exogenous *Cry1Ac* gene (Adamczyk and Sumerford, 2001). This indicates that there are few endogenous factors that affect the accumulation of exogenous proteins, which can function independently of the endogenous metabolic pathways of recipient crops (Palma et al., 2014; Melo et al., 2016). However, endogenous proteins encoded by the overexpressed endogenous genes are usually regulated in complex networks with functional redundance or replaceable pathways and are difficult to confer the desirable phenotypes significantly (Winkel, 2004; Fiehn and Weckwerth, 2010; Wan et al., 2017).

### 4.3 Higher enzyme activity of proteins encoded by exogenous genes

The result of the enzyme reaction assay showed that the activities of the ADH enzyme in the three T_3_ lines transformed by the exogenous *ZmADH* gene and two T_3_ lines transformed by the exogenous *EcADH* gene were greatly significantly higher than that of the wild-type control (**Figure 6A**). Moreover, the average ADH enzyme activity in the three T_3_ lines transformed by the exogenous *ZmADH* gene and in the three T_3_ lines transformed by the exogenous *EcADH* gene were both significantly higher than the average ADH enzyme activity in the three T_3_ lines transformed by the endogenous *AtADH* gene (**Figure 6B**). This result indicated that the enzyme activity of the proteins encoded by exogenous genes was higher than that of the proteins encoded by endogenous genes.

### 4.4 Limitaions to be improved

One of the T_3_ lines transformed by the exogenous *EcADH* gene did not show a significant increase in enzyme activity (**Figure 6A**), although its relative expression level of and accumulation of the EcADH protein encoded by the transformed exogenous *EcADH* gene were significantly increased (**Figures 3B**, **5A**). This exception should be investigated further. In addition, more homozygous transgenic lines should be transformed and screened for elimination of potential biases probably caused by variable T-DNA integration sites or operational deviation on specific methodologies like RT-qPCR, ELISA, and enzyme activity assay.

## 5 Conclusion

The relative expression levels of the transformed exogenous ZmADH and EcADH genes were ten or tens of times higher than that of the transformed endogenous AtADH gene. The accumulation levels of the proteins encoded by the transformed ZmADH and EcADH were significantly higher than that encoded by the transformed endogenous AtADH. The ADH activities of the T_3_ lines transformed by the exogenous ZmADH and EcADH were also significantly higher than that transformed by the endogenous AtAHD as well as the wild-type control. According to these results, along with database and literature searches, it can be concluded that exogenous genes are more conducive to transgenic improvement of crop varieties. It is suggested that more attention should be paid to the transformation of exogenous genes from distant species for transgenic improvement of crops, especially for abiotic and biotic tolerance as well as mechanism pathways.

## Data Availability

The raw data supporting the conclusions of this article will be made available by the authors, without undue reservation.
