# Supplementary material for: Expression evaluation of exogenous and endogenous alcohol dehydrogenase genes in transgenic Arabidopsis
Source: Front Plant Sci. 2025 Jan 22;15:1476754. doi: 10.3389/fpls.2024.1476754 (PMC11794282; doi:10.3389/fpls.2024.1476754)
Supplement: Supplementary file 1 [file Supplementaryfile1.docx]

Supplementary Material

# Table S1 Homologous recombination primers for CDS cloning of the *ADH* genes

| Gene | Sequence | Amplified length |
| --- | --- | --- |
| *AtADH* | 5'-gagaacacgggggactctagaATGTCTACCACCGGACAGATTATTC-3' | 1140 bp |
|  | 5'-tccctcgaggagctctctagaAAGCACCCATGGTGATGATGC-3' |  |
| *ZmADH* | 5'-gagaacacgggggactctagaATGGCGACCGCGGGGAAG-3' | 1140 bp |
|  | 5'-tccctcgaggagctctctagaAGTTCTCCATGCGGATGATGC-3' |  |
| *EcADH* | 5'-gagaacacgggggactctagaATGAAGGCTGCAGTTGTTACGA-3' | 1011 bp |
|  | 5'-tccctcgaggagctctctagaAGCGGCGGAAATCAATCAC-3' |  |
| * The lowercase and underlined letters show the homologous sequences of expression vector pBI121-cMycNY and the restriction site of *Xbal* Ⅰ, respectively. | | |


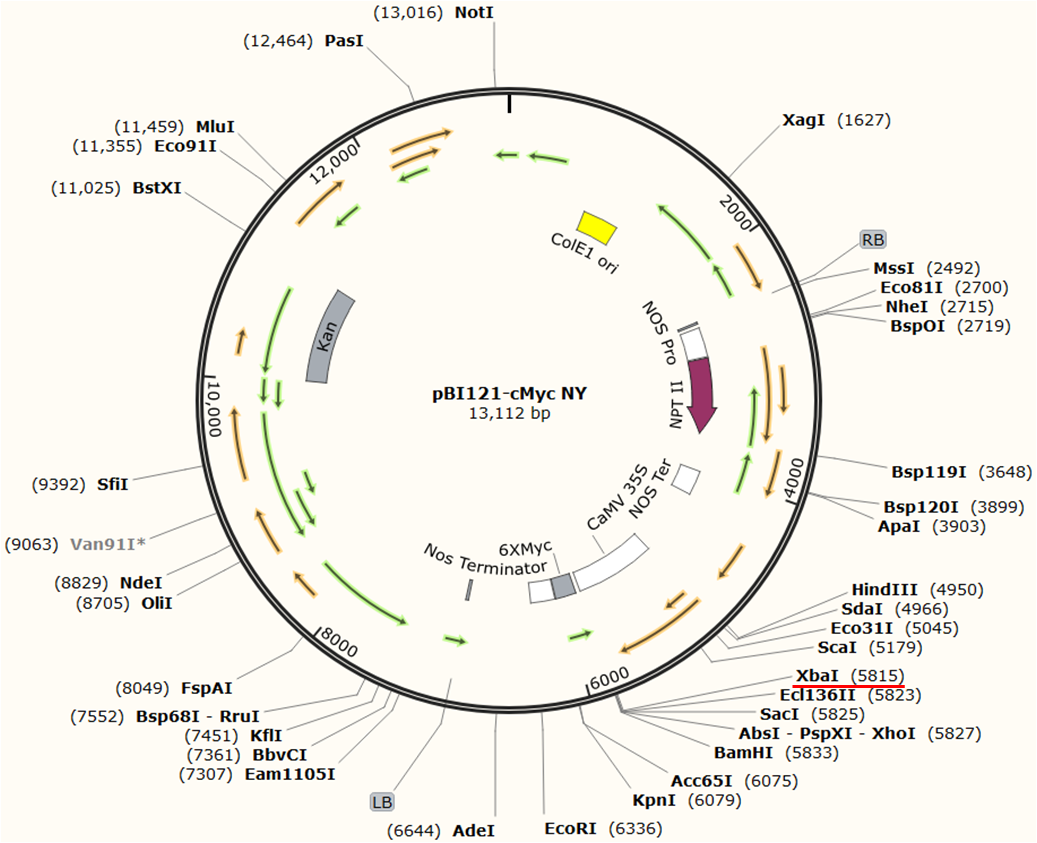


# Figure S1 Dicotyledonous expression vector pBI121-cMyc-NY.

# Table S2 Amplification primers for T-DNA flanking sequences

| Lines | Border | Primer sequence | Amplified length |
| --- | --- | --- | --- |
| AtADH-3 | Left | 5'-TCACTGGCCGTCGTTTTACA-3' | 1219 bp |
|  |  | 5'-GTCTTCGCAAGTGAGAGGCT-3' |  |
|  | Right | 5'-AGCTTCGTCTTCGCAAGTGA-3' | 1057 bp |
|  |  | 5'-ACGTTGAAGGAGCCACTCAG-3' |  |
|  | Left | 5'-TGGAGACACAAAGGAGCACA-3' | 1062 bp |
|  |  | 5'-GCGCTTTACTGGCACTTCAG-3' |  |
|  | Right | 5'-TTCAGTGACAACGTCGAGCA-3' | 1210 bp |
|  |  | 5'-CACCAACGACCCTAGGTCAA-3' |  |
| AtADH-4 | Left | 5'-TGCCTAAGCTGACTAATCGCA-3' | 896 bp |
|  |  | 5'-TCACTGGCCGTCGTTTTACA-3' |  |
|  | Right | 5'-TCGTGACTGGGAAAACCCTG-3' | 1242 bp |
|  |  | 5'-TCGTGGGATCGTGTTTGTGAT-3' |  |
|  | Left | 5'-ATCTGATGCCTCGACCCCTA-3' | 1022 bp |
|  |  | 5'-CAAGCTCTAAATCGGGGGCT-3' |  |
|  | Right | 5'-GCTGTATGCGTTGGTGCAAT-3' | 1204 bp |
|  |  | 5'-ATGCGAGCAAAACGAAGCAA-3' |  |
| AtADH-11 | Left | 5'-GAAAGCCATGCGCATTGAGG-3' | 1120 bp |
|  |  | 5'-GATCCAGATCCGGTGCAGATT-3' |  |
|  | Right | 5'-TCACTGGCCGTCGTTTTACA-3' | 1175 bp |
|  |  | 5'-TGTAGCTCAATGGGGACTGC-3' |  |
| AtADH-17 | Left | 5'-GAACACCATTTGCCAGGGGA-3' | 1049 bp |
|  |  | 5'-GCCAGTGAATTCCCGATCT-3' |  |
|  | Right | 5'-CGTATGATTCTCCGCCAGCA-3' | 1212 bp |
|  |  | 5'-TGGCTTTGCAGATTTGTGAACAT-3' |  |
|  | Left | 5'-TGGGTCAGGATTAGGGTCGT-3' | 1055 bp |
|  |  | 5'-CGGTCATTTCGAACCCCAGA-3' |  |
|  | Right | 5'-TCACTGGCCGTCGTTTTACA-3' | 1053 bp |
|  |  | 5'-TTTTGCGGTGTCGCTTTCAG-3' |  |
| AtADH-18 | Left | 5'-TTTGGCTGGATTTGTGGTGC-3' | 1029 bp |
|  |  | 5'-ACCATTAGCAAGGCCGGAAA-3' |  |
|  | Right | 5'-TCGTGACTGGGAAAACCCTG-3' | 1033 bp |
|  |  | 5'-CAAAGACCACCTACCTTGTCTGA-3' |  |
| ZmADH-1 | Left | 5'-CCAAGTTCCATCAGAGCCCAA-3' | 1075 bp |
|  |  | 5'-TTGAATCCTGTTGCCGGTCT-3' |  |
|  | Right | 5'-GAACCTGCGTGCAATCCATC-3' | 1377 bp |
|  |  | 5'-GAGCTTTTCTGTCCAGGGAG-3' |  |
| ZmADH-2 | Left | 5'-CTCACCTGAATCACACCGCT-3' | 1048 bp |
|  |  | 5'-TGCCGGTCTTGCGATGATTA-3' |  |
|  | Right | 5'-TCGTGACTGGGAAAACCCTG-3' | 1223 bp |
|  |  | 5'-TCCACACAATGCACACAACC-3' |  |
| ZmADH-4 | Left | 5'-ACGTGCGTGAATGGTGATGTA-3' | 1114 bp |
|  |  | 5'-CCGCTCAGAAGAACTCGTCA-3' |  |
|  | Right | 5'-TCACTGGCCGTCGTTTTACA-3' | 1129 bp |
|  |  | 5'-TCCAGCCCCTAGTCACGATT-3' |  |
| EcADH-1 | Left | 5'-TGTTTCCGAGCCATCAACCA-3' | 1039 bp |
|  |  | 5'-ACGGTTTTTCGCCCTTTGAC-3' |  |
|  | Right | 5'-TTTGCTCCATGGTGAGGTCG-3' | 1468 bp |
|  |  | 5'-ACACGCATTCTTCCGCCTAA-3' |  |
|  | Left | 5'-GTATGGCGGTGTCAGGTGAA-3' | 1162 bp |
|  |  | 5'-TCACTGGCCGTCGTTTTACA-3' |  |
|  | Right | 5'-TGCCGGTCTTGCGATGATTA-3' | 1449 bp |
|  |  | 5'-ACATGAGCCTCTCCGGTTTG-3' |  |
| EcADH-2 | Left | 5'-TGACCGTATTACATGCTGATCCA-3' | 1139 bp |
|  |  | 5'-TCGTGACTGGGAAAACCCTG-3' |  |
|  | Right | 5'-TGATGGTTCACGTAGTGGGC-3' | 1000 bp |
|  |  | 5'-CTTCTTGATGTTCCGCGTCG-3' |  |
|  | Left | 5'-ATTCTGGGCAGTCTCGAACC-3' | 1263 bp |
|  |  | 5'-TCGTGACTGGGAAAACCCTG-3' |  |
|  | Right | 5'-TTGAATCCTGTTGCCGGTCT-3' | 1118 bp |
|  |  | 5'-GTGTGAATGCGGAAGGGAGA-3' |  |
| EcADH-4 | Left | 5'-GGGACTATGTTGTGGTATCCTCC-3' | 1056 bp |
|  |  | 5'-TCACTGGCCGTCGTTTTACA-3' |  |
|  | Right | 5'-GTCGTGACTGGGAAAACCCT-3' | 1248 bp |
|  |  | 5'-CTGGCTCAAGAATGTGTCGC-3' |  |
| EcADH-5 | Left | 5'-CACTCCACAGCGATTCTGGT-3' | 1284 bp |
|  |  | 5'-TCACTGGCCGTCGTTTTACA-3' |  |
|  | Right | 5'-AGATTGTCGTTTCCCGCCTT-3' | 1218 bp |
|  |  | 5'-CCACATGGCCTTACTTTGGC-3' |  |
| EcADH-9 | Left | 5'-ACGTTCCTTGGTGTGACAGA-3' | 1202 bp |
|  |  | 5'-TGATGGTTCACGTAGTGGGC-3' |  |
|  | Right | 5'-TCACTGGCCGTCGTTTTACA-3' | 1283 bp |
|  |  | 5'-ATCAAAAGCTTGTCCGCAGC-3' |  |
| EcADH-13 | Left | 5'-ATGCGATTTTGGGGAGAGCA-3' | 1016 bp |
|  |  | 5'-CACTCAACCCTATCTCGGGC-3' |  |
|  | Right | 5'-TTGAATCCTGTTGCCGGTCT-3' | 1484 bp |
|  |  | 5'-GGGGAAGATCGAGTGGTTCG-3' |  |
|  | Left | 5'-TCCTTGCCGACATTCAAAAGA-3' | 1005 bp |
|  |  | 5'-TGTGCGTCATCCCTTACGTC-3' |  |
|  | Right | 5'-TGATGGTTCACGTAGTGGGC-3' | 1156 bp |
|  |  | 5'-TTGGGGATTCAACACGCTCT-3' |  |

# Table S3 Primers for RT-qPCR

| Gene | Sequence | Amplified length |
| --- | --- | --- |
| *AtADH* | 5'-TGTGGGGAGTGTCGTCATTG-3' | 154 bp |
|  | 5'-TGAACGTGGAAGTCCCAAGG-3' |  |
| *ZmADH* | 5'-TGCTCAGGATCAACACCGAC-3' | 191 bp |
|  | 5'-ACCACAGCTAAGGACGCAAA-3' |  |
| *EcADH* | 5'-GGTATTGGTGTGGTGGCAGA-3' | 143 bp |
|  | 5'-TTAACTGAACGGCAGAGCGT-3' |  |
| *AtUBQ5* | 5'-CGTACCCTCGCCGACTACAA-3' | 188 bp |
|  | 5'-CGCTGAACCTTTCCAGATCCA-3' |  |


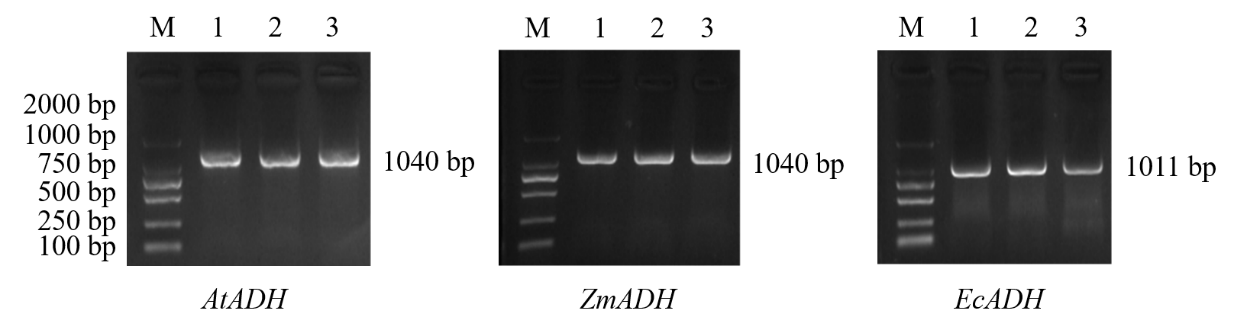


# Figure S2 Electrophoresis of amplified CDSs of genes *AtADH*, *ZmADH*, and *EcADH*. M: DNA marker DL2000, Lanes 1, 2, and 3: amplified CDSs of genes *AtADH*, *ZmADH*, and *EcADH*.


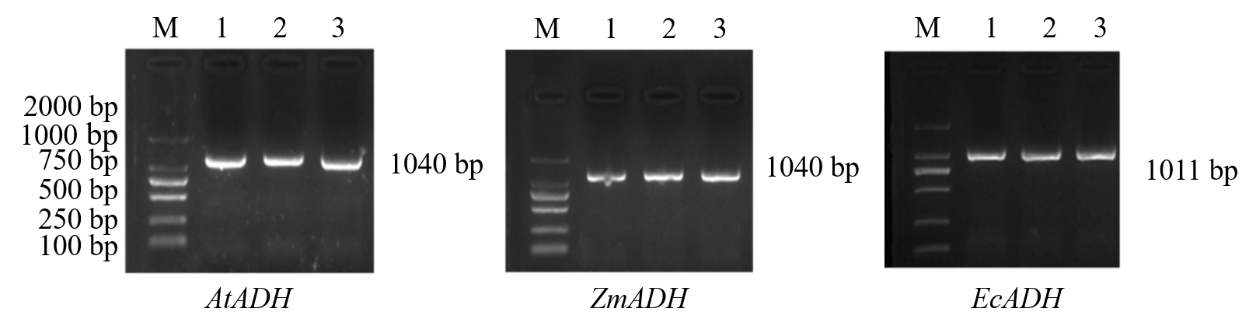


# Figure S3 Electrophoresis of bacterial PCR amplified CDSs of genes *AtADH*, *ZmADH*, and *EcADH* constructed into dicotyledonous expression vector pBI121-cMyc-NY and transformed into *Agrobacterium tumefaciens*. M: DNA marker DL2000, Lanes 1, 2, and 3: amplified CDSs of genes *AtADH*, *ZmADH*, and *EcADH*.

#
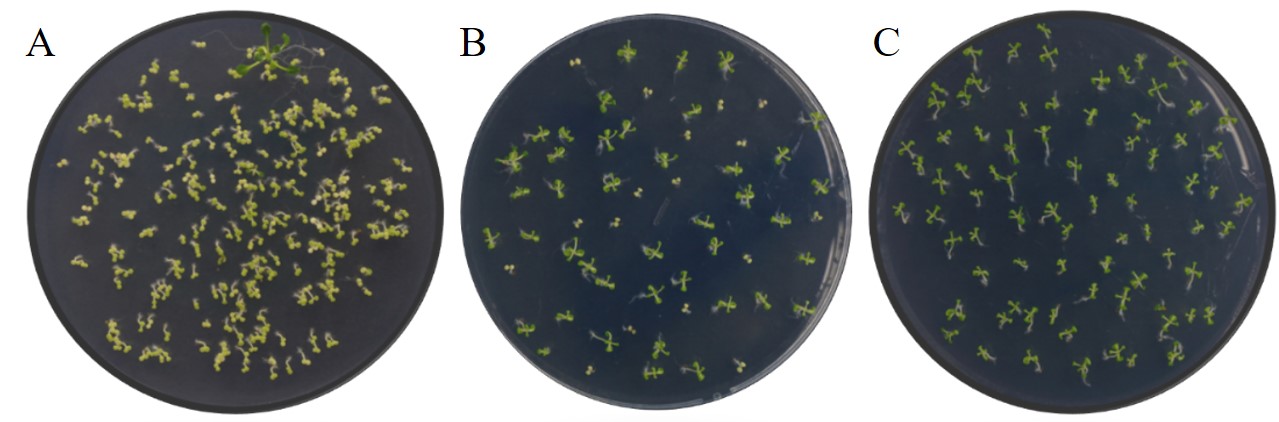


**Figure S4 Resistant screening of transgenic lines.** A: T_0_, B: T_1_, and C: T_3_.

# Table S4 Integration sites of T-DNA identified by resequencing and alignment of genome of T_3_ lines

| Lines | Border | Integration site | T-DNA  cleavage site | Number of  filled base | Direction | Inserted length |
| --- | --- | --- | --- | --- | --- | --- |
| AtADH-3 | Left | Chr1:8590480 bp | 6980 bp | 0 bp | Forward | 4993 bp |
|  | Right | Chr1: 8590598 bp | 1987 bp | 0 bp |  |  |
|  | Left | Chr2: 18278069 bp | 1600 bp | 0 bp | Forward | 12236 bp |
|  | Right | Chr2:18278115 bp | 2476 bp | 5 bp |  |  |
| AtADH-4 | Left | Chr1:24661902 bp | 6970 bp | 2 bp | Reverse | 13120 bp |
|  | Right | Chr1:24661911 bp | 6962 bp | 23 bp |  |  |
|  | Left | Chr5:6014054 bp | 6961 bp | 6 bp | Reverse | 4487 bp |
|  | Right | Chr5:6014068 bp | 2474 bp | 38 bp |  |  |
| AtADH-11 | Left | Chr5:2751567 bp | 2586 bp | 32 bp | Forward | 4404 bp |
|  | Right | Chr5:2751582 bp | 6990 bp | 1 bp |  |  |
| AtADH-17 | Left | Chr2:9478424 bp | 6963 bp | 0 bp | Reverse | 4546 bp |
|  | Right | Chr2:9478450 bp | 2417 bp | 0 bp |  |  |
|  | Left | Chr5:26100184 bp | 2930 bp | 0 bp | Forward | 4024 bp |
|  | Right | Chr5:26100236 bp | 6954 bp | 0 bp |  |  |
| AtADH-18 | Left | Chr1:29335302 bp | 4200 bp | 0 bp | Forward | 2778 bp |
|  | Right | Chr1:29335335 bp | 6978 bp | 0 bp |  |  |
| ZmADH-1 | Left | Chr3:2531833 bp | 6980 bp | 1 bp | Reverse | 4500 bp |
|  | Right | Chr3:2545407 bp | 2480 bp | 31 bp |  |  |
| ZmADH-2 | Left | Chr4:12012734 bp | 6955 bp | 5 bp | Forward | 13131 bp |
|  | Right | Chr4:12012749 bp | 6974 bp | 0 bp |  |  |
| ZmADH-4 | Left | Chr1:25784401 bp | 2701 bp | 50 bp | Forward | 4279 bp |
|  | Right | Chr1:25784420 bp | 6980 bp | 53 bp |  |  |
| EcADH-1 | Left | Chr1:29263031 bp | 6978 bp | 4 bp | Reverse | 12016 bp |
|  | Right | Chr1:29263163 bp | 5882 bp | 7 bp |  |  |
|  | Left | Chr4:9329207 bp | 6974 bp | 0 bp | Reverse | 13115 bp |
|  | Right | Chr4:9320293 bp | 6971 bp | 19 bp |  |  |
| EcADH-2 | Left | Chr3:19493571 bp | 6959 bp | 0 bp | Reverse | 13113 bp |
|  | Right | Chr3:19493578 bp | 6958 bp | 37 bp |  |  |
|  | Left | Chr3:20753834 bp | 6976 bp | 24 bp | Forward | 13117 bp |
|  | Right | Chr3:20753862 bp | 6985 bp | 0 bp |  |  |
| EcADH-4 | Left | Chr3:21462132 bp | 6961 bp | 30 bp | Forward | 13124 bp |
|  | Right | Chr3:21462139 bp | 6973 bp | 3 bp |  |  |
| EcADH-5 | Left | Chr5:22345445 bp | 6966 bp | 8 bp | Forward | 11887 bp |
|  | Right | Chr5:22345484 bp | 8191 bp | 0 bp |  |  |
| EcADH-9 | Left | Chr5:21149754 bp | 6980 bp | 6 bp | Reverse | 13114 bp |
|  | Right | Chr5:21149802 bp | 6978 bp | 5 bp |  |  |
| EcADH-13 | Left | Chr2:8794301 bp | 6988 bp | 0 bp | Reverse | 13129 bp |
|  | Right | Chr2:8794341 bp | 6971 bp | 0 bp |  |  |
|  | Left | Chr3:6704557 bp | 4938 bp | 0 bp | Forward | 2012 bp |
|  | Right | Chr3:6704616 bp | 6950 bp | 11 bp |  |  |

# Table S5 Integration sites of T-DNA identified by sequencing and alignment of their flanking sequences

| Lines | Border | Aligned sequence | E_value | Similarity |
| --- | --- | --- | --- | --- |
| AtADH-3 | Left | Chr1 | 0.0 | 100.00% |
|  |  | T-DNA | 0.0 | 100.00% |
|  | Right | T-DNA |  |  |
|  |  | Chr 1 |  |  |
|  | Left | Chr2 |  |  |
|  |  | T-DNA |  |  |
|  | Right | T-DNA | 0.0 | 99.65% |
|  |  | Chr 2 | 0.0 | 100.00% |
| AtADH-4 | Left | Chr5 | 0.0 | 99.30% |
|  |  | T-DNA | 0.0 | 100.00% |
|  | Right | T-DNA | 7.00E^-103^ | 99.01% |
|  |  | Chr 5 | 0.0 | 98.90% |
|  | Left | Chr1 | 3.40E^-44^ | 94.80% |
|  |  | T-DNA | 0.0 | 97.71% |
|  | Right | T-DNA | 0.0 | 94.80% |
|  |  | Chr 1 | 6.70E^-61^ | 100.00% |
| AtADH-11 | Left | Chr5 | 4.30E^-61^ | 100.00% |
|  |  | T-DNA | 1.00E^-90^ | 95.96% |
|  | Right | T-DNA | 0.0 | 99.83% |
|  |  | Chr 5 | 0.0 | 100.00% |
| AtADH-17 | Left | Chr2 |  |  |
|  |  | T-DNA |  |  |
|  | Right | T-DNA |  |  |
|  |  | Chr 2 |  |  |
|  | Left | Chr5 | 0.0 | 100.00% |
|  |  | T-DNA | 0.0 | 100.00% |
|  | Right | T-DNA | 0.0 | 100.00% |
|  |  | Chr 5 | 0.0 | 100.00% |
| AtADH-18 | Left | Chr1 | 0.0 | 100.00% |
|  |  | T-DNA | 0.0 | 100.00% |
|  | Right | T-DNA | 0.0 | 100.00% |
|  |  | Chr 1 | 2.90E^-40^ | 93.80% |
| ZmADH-1 | Left | Chr3 | 2.40E^-73^ | 100.00% |
|  |  | T-DNA | 0.0 | 99.88% |
|  | Right | T-DNA | 0.0 | 99.85% |
|  |  | Chr 3 | 0.0 | 100.00% |
| ZmADH-2 | Left | Chr4 | 9.20E^-104^ | 100.00% |
|  |  | T-DNA | 0.0 | 99.50% |
|  | Right | T-DNA | 0.0 | 100.00% |
|  |  | Chr 4 | 0.0 | 100.00% |
| ZmADH-4 | Left | Chr1 | 6.10E^-68^ | 100.00% |
|  |  | T-DNA | 0.0 | 99.89% |
|  | Right | T-DNA | 5.00E^-168^ | 100.00% |
|  |  | Chr 1 | 0.0 | 99.50% |
| EcADH-1 | Left | Chr 1 | 0.0 | 97.6 0% |
|  |  | T-DNA | 6.00E^-108^ | 100.00% |
|  | Right | T-DNA | 2.00E^-78^ | 100.00% |
|  |  | Chr 1 | 0. | 93.60% |
|  | Left | Chr 4 | 0.0 | 100.00% |
|  |  | T-DNA | 0.0 | 99.83% |
|  | Right | T-DNA | 0.0 | 99.75% |
|  |  | Chr 4 | 0.0 | 100.00% |
| EcADH-2 | Left | Chr3 | 3.90E^-108^ | 100.00% |
|  |  | T-DNA | 0.0 | 99.30% |
|  | Right | T-DNA | 5.00E^-158^ | 99.66% |
|  |  | Chr 3 | 0.0 | 100.00% |
|  | Left | Chr 3 | 2.30E^-60^ | 94.70% |
|  |  | T-DNA | 0.0 | 99.82% |
|  | Right | T-DNA | 0.0 | 98.69% |
|  |  | Chr 3 | 1.10E^-67^ | 100.00% |
| EcADH-4 | Left | Chr3 | 0.0 | 100.00% |
|  |  | T-DNA | 0.0 | 99.54% |
|  | Right | T-DNA | 0.0 | 99.65% |
|  |  | Chr 3 | 0.0 | 100.00% |
| EcADH-5 | Left | Chr5 | 0.0 | 100.00% |
|  |  | T-DNA | 0.0 | 100.00% |
|  | Right | T-DNA | 0.0 | 100.00% |
|  |  | Chr 5 | 0.0 | 100.00% |
| EcADH-9 | Left | Chr 5 | 0.0 | 100.00% |
|  |  | T-DNA | 7.00E^-163^ | 100.00% |
|  | Right | T-DNA | 0.0 | 100.00% |
|  |  | Chr 5 | 0.0 | 99.80% |
| EcADH-13 | Left | Chr 2 | 0.0 | 100.00% |
|  |  | T-DNA | 1.00E^-104^ | 99.51% |
|  | Right | T-DNA |  |  |
|  |  | Chr 2 |  |  |
|  | Left | Chr 3 |  |  |
|  |  | T-DNA |  |  |
|  | Right | T-DNA | 1.00E^-154^ | 99.32% |
|  |  | Chr 3 | 0.0 | 100.00% |
